# Removal of cyanobacterial harmful algal blooms (HABs) from contaminated local park lake using *Ganoderma lucidum* mycelial pellets

**DOI:** 10.1016/j.heliyon.2024.e41205

**Published:** 2024-12-15

**Authors:** Zarimah Mohd Hanafiah, Anggita Rahmi Hafsari, Malini Elango, Zul Ilham, Febri Doni, Wan Hanna Melini Wan Mohtar, Yusufjon Gafforov, Yong Jie Wong, Khairul Nizam Abdul Maulud, Nor Hidayah Ismail, Mohd Yusmiaidil Putera Mohd Yusof, Wan Abd Al Qadr Imad Wan-Mohtar

**Affiliations:** a Functional Omics and Bioprocess Development Laboratory, Institute of Biological Sciences, Faculty of Science, Universiti Malaya, Kuala Lumpur, 50603, Malaysia; b Department of Civil Engineering, Faculty of Engineering and Build Environment, Universiti Kebangsaan Malaysia (UKM), Bangi, 43600, Malaysia; c Biomass Energy Laboratory, Faculty of Science, Institute of Biological Sciences, Universiti Malaya, 50603, Kuala Lumpur, Malaysia; d Department of Biology, Faculty of Mathematics and Natural Sciences, Universitas Padjadjaran, Jatinangor, West Java, 45363, Indonesia; e Central Asian Center for Development Studies, New Uzbekistan University, 100000, Tashkent, Uzbekistan; f Department of Bioenvironmental Design, Faculty of Bioenvironmental Sciences, Kyoto University of Advanced Science, Kyoto, 606-8501, Japan; g Research Grant Management Division, Department of Research Management, Universiti Malaya, 50603, Kuala Lumpur, Malaysia; h Institute of Pathology, Laboratory and Forensic Medicine (I-PPerForM), Universiti Teknologi MARA Selangor, Sungai Buloh, Selangor, Malaysia

**Keywords:** *Ganoderma lucidum*, Bio-flocculation, Blue-green algae, Water treatment, Cyanobacteria

## Abstract

Eutrophication and hypereutrophication in lakes foster harmful blue-green algal blooms, which pose a significant threat to the ecological health of freshwater reservoirs. This study investigated the effectiveness of the bio-flocculation approach using the *Ganoderma lucidum* fungus strain BGF4A1 to remove these harmful blooms, specifically targeting cyanobacterial species like *Microcystis* PCC-7914. Key flocculation parameters, cyanobacterial concentrations, adsorption kinetics, and pellet morphology were explored in this research. Our results demonstrate that *G. lucidum* can effectively remove up to 93.70 % of cyanobacteria (measured as chlorophyll-*a* absorbance), 75.28 % of chemical oxygen demand (COD), and 92.09 % of total suspended solids (TSS) under optimal conditions: an initial pH of 4, 1 % fungal volume (w/v), 48 h of contact time, and 100 rpm agitation at room temperature. Microscopic examination of water samples before and after treatment confirmed a significant reduction in cyanobacterial colonies, indicating the death or decline of the targeted organisms. Morphological analysis using field emission scanning electron microscopy (FESEM) revealed that *Microcystis* cells were deposited on the hyphae of the *G. lucidum* pellets, in contrast to the smooth surface of control pellets. These novel culture technologies show great promise as bio-flocculating agents for removing blue-green algae and potentially be adapted for microalgae harvesting in biodiesel production.

## Highlights

• *G. lucidum* can effectively remove up to 93.70 % of cyanobacteria, 75.28 % of COD, and 92.09 % of TSS.
• Optimal conditions for flocculation were identified at pH 4, 1 % inoculum, 48 h contact time.
• Microscopic examination confirmed a significant reduction in cyanobacterial colonies.
• Scanning electron image showed cyanobacterial cells adsorbed on the fungal hyphae.

## 1 Introduction

Cyanobacterial blooms, also known as harmful algal blooms (HABs) and blue-green algae, are the most prevalent and widely studied type of harmful algal bloom in many freshwater environments like lakes and ponds. *Microcystis* sp. is the most commonly reported species of cyanobacteria, which can be found in numerous regions [[1], [2], [3]]. Cyanobacterial HABs are characterized by rapid and excessive accumulations in aquatic environments. These blooms are typically fueled by an excess of nutrients like nitrogen and phosphorus, a phenomenon known as eutrophication, combined with favorable environmental conditions such as warm temperatures, calm water conditions, and abundant sunlight [4].

Cyanobacterial HABs are detrimental to the health of the water bodies due to their accumulation on the surface water column and the rotting of stagnant biomass, leading to problems related to color, taste, and odor. Moreover, cyanobacterial HABs pose a significant threat to aquatic ecosystems worldwide by disrupting the zooplankton community, degrading habitats, and altering the stability of these ecosystems [5]. Of the utmost concern, cyanobacterial HABs endanger both animal and human health through their production of various toxins. Multiple cyanobacterial genera, including *Microcystis*, *Anabaena*, *Anabaenopsis*, and *Planktothrix*, are capable of producing a wide range of toxins, including microcystins, saxitoxins, anatoxins, and cylindrospermopsins [4]. These toxins primarily target the nervous tissue, brain, lungs, liver, and kidneys [6]. Additionally, the toxins produced by cyanobacterial HABs, particularly cylindrospermopsins and microcystins, can directly affect the immune system, stomach, esophagus, the small intestine, colon, and gastrointestinal tract when ingested [7]. Humans can also become infected and ill from poisoning by cyanobacterial toxins through the consumption of contaminated shellfish [8]. Furthermore, there is a survey concerning the cyanotoxins contaminated water can also lead to cardiovascular disease (CVD) [9]. Prolonged exposure to the contaminated water can increase the chances of serious health problems, including CVD [10,11]. However, the adverse CVD effects are expected due to indirect effects arising from damage in other organs [9].

Strategies for addressing cyanobacterial HABs in lake systems are crucial. While reducing nutrient content in the water column is essential, it can be a slow mitigation process [12]. Therefore, water treatment is often necessary to accelerate mitigation. This typically involves physical, chemical, and biological interventions (Table 1). Physical treatments include harvesting floating cells using filters, pumps, and barriers, as well as employing ultrasound, adsorption with activated carbon or clay, and irradiation. Chemical treatments encompass algicides, algal inhibitors, hydrogen peroxide, and ozone [[13], [14], [15]]. Physical and chemical treatment methods have been used for a longer period compared to biological methods [16]. However, physical methods are labor-intensive and costly, especially for large water bodies. Although chemical methods are quick and effective, they can lead to secondary pollution [17,18].

**Table 1:** The type of algae control technology, application and characteristic.

Treatment Categories	Treatment Types	Methods	Explanations	References
Physical	Adsorption	Using biosorbent such as activated carbon and clay to trap/floc then precipitate.	Simple and economical equipment, but depends on equipment and power energy.	[18]
	Ultrasound (US)	The suspended biomass cells were destroyed/inactivated by the US effect.	US equipment is simple but, has higher US intensity leads to higher energy consumption and effect to aquatic organisms.	[19]
	Filter	Including sand filtration and membrane to remove algae without breaking the cells.	Equipment is simple but require energy and high algae cell concentrations may clog the filter.	[20]
Chemical	Hydrogen Peroxide	Inhibiting algae growth, thus stopping the bloom from spreading.	High efficiency but costly and may produce by-products via through the oxidation process.	[21]
	Chlorine	Chemical reaction that stops the algae growth	Simple and feasible, however, cost is high and possibly contain by product and harsh operating conditions.	[14]
	Ozone	Pre-oxidation to coagulate the algae biomass	-High efficiency, but costly and possibly produce by product via oxidation process.	[22]
Biological	Aquatic animal (predation)	Control by releasing fish, shellfish and other suitable aquatic animals.	Simple and sustainable, but high cost and complicated maintenance.	[23]
	Biological floating bed	Aquatic plant is used to remove nutrient focusing on nitrogen and phosphorus.	Landscaping water, however, controlling the growth for huge water system is difficult.	[24]
	Microorganism	Introduce algae-degrading or algae-adsorbing microorganism such as bacteria and fungi.	Microorganism species are abundant and inexpensive, but upscaling requires further research.	[25]

Biological treatment for of HAB remediation utilizes living organisms or natural processes to control algal blooms in aquatic ecosystems through mechanisms such as adsorption [[26], [27], [28]], predation [23], and degradation [29]. Traditional biological methods involving predation by aquatic animals and plants face challenges due to geographical constraints, lengthy treatment cycles, and potential disruptions to ecosystem stability [30]. Recent technological advancements, such as multi-soil-layering (MSL) and constructed wetlands, have emerged as promising techniques for wastewater treatment. MSL systems, as demonstrated by Aba et al. [31], and Mugani et al. [32], offer high cyanobacteria removal rates (52–99 %). However, careful selection and combination of soil layers are crucial to ensure optimal system performance. Constructed wetlands, as studied by Bavithra et al. [33], can also achieve high cyanobacteria removal rates (up to 99 %) within a one-week cycle, but they require significant land area.

Another type of biological treatments such as using microbial treatments offer promising potential for HAB remediation. Microbial treatment offers cost-effectiveness, applicability in controlled settings with shorter treatment cycles, high efficiency, and additional benefits beyond HAB inhibition, such as breaking down organic matter and improving water quality [13,34]. While current research primarily focuses on the mechanisms of bacterial action [35], there is a growing interest in the use of fungi, particularly because of their versatility and unique enzymatic capabilities.

Fungi species such as white rot fungi (WRF), known for their efficient degradation of a wide range of pollutants including organic compounds [36,37], pharmaceuticals [38], dyes [39], heavy metals, and micropollutants, have also shown potential in controlling algal blooms. Studies have demonstrated that fungi can control HABs through several mechanisms, including nutrient competition (fungi compete with algae in utilizing nutrients such as nitrogen and phosphorus, limiting their growth), allelopathy (secondary metabolites from fungi to inhibit HAB growth), antagonism (directly kill the algal cell), adsorption and flocculation [13]. As natural organisms, fungi offer a more environmentally sustainable option compared to chemical treatments. They are relatively low-cost to cultivate and can be applied to a wide range of aquatic environments [40]. Previous research has shown promising potential for fungi in HAB remediation.

For instance, Nie et al. [27], bio-flocculated more than 90 % of *Microcystis aeruginosa* using the WRF fungus *Aspergillus oryzae* in conditions of 11 g/L fungus dosage, 5 h of flocculation time, in 100 rpm rotation speed and optimal pH of 4. Another study, Wang et al. [41], used the WRF fungus *Phanerochaete chrysosporium* to lyse 85.48 % of algal cells (Turpin) for 48 h of treatment and using similar WRD culture, Zeng et al. [17], used to inhibit algae (*Cryptomonas obovata*, *Oscillatoria* sp., and *Scenedesmus quadricauda*) by method of co-culturing found that the chlorophyll-*a* content reduces up to 84.16 % after 48 h of treatment. While, Hu et al. [25], studies another mechanism of WRF fungi (*Trametes versicolor*) treating algae (*Amphidinium carterae*) as algaecides and results show that fungi's enzymes enhance the antialgal property. These studies demonstrate the promising of using fungi in bio-flocculating, lysing algal cells, reducing toxin production, and regulating algal growth dynamics, thus sustainably mitigating HABs in reservoirs.

*Ganoderma lucidum* is one of the WRF cultures widely used in wastewater treatment in removal pollutant. It can break down complex pollutants in wastewater, such as heavy metal through adsorption [42], pharmaceutical compound via enzyme degradation [38], organic compound such as chemical oxygen demand (COD) [43] and dye removal through adsorption [44]. Till to date, the applications of *Ganoderma lucidum* is not reported yet in literature, however, this strain has been used as microalgae harvesting agent, such as *Chlorella* sp [45,46]. Due to promising in as the harvesting microalgae agent, water treatment agent, locally available and easy for cultivation [47,48], the *Ganoderma lucidum* is promising candidate to be explored to treat harmful algae.

Given the promising potential of Ganoderma species in water treatment applications, this study aims to explore the potential of the medicinal fungus *Ganoderma lucidum* for HAB removal. The objectives of this research are to (a) assess the water quality and cyanobacterial content of former mining lakes, (b) investigate the potential of bio-flocculating cyanobacterial HABs using *G. lucidum* pellets, and (c) characterize *G. lucidum* after cyanobacterial HAB treatment. To evaluate the lake water categories and assess the potential of *G. lucidum* treatment, the lake water trophic index was calculated and morphological image analysis of the cells was conducted. The proposed nature-based treatment method using *G. lucidum* pellets aligns well with the current paradigm, supporting the broader goals of enhancing treatment efficiency, cost-effectiveness, energy efficiency, and sustainability in wastewater management. Additionally, the findings of this research could resonate with public sentiment, as there is a growing awareness of and demand for environmentally friendly wastewater treatment solutions.

## 2 Materials and methods

### 2.1 Description of the locations and water samples

**Fig. 1:**
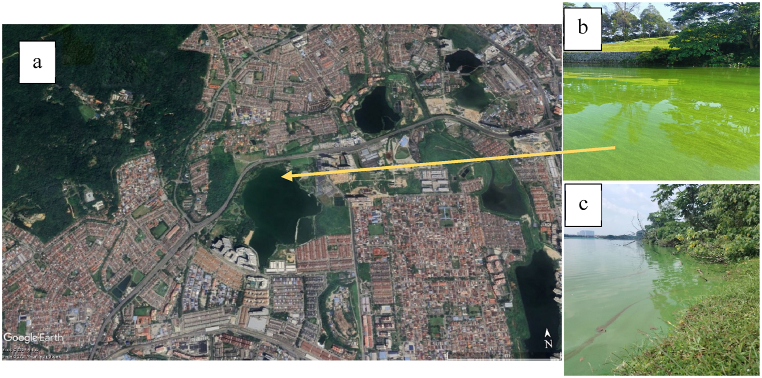
The study location (a) located in densely populated area and (b, c) the cyanobacterial bloom.

Cyanobacteria-contaminated water was collected from an urban residential park lake located in Klang Valley, in the state of Selangor, Malaysia as shown in Fig. 1-a. Surrounded by densely populated areas, this popular recreational lake is a hub for outdoor activities such as jogging, cycling, kite flying, picnicking, and enjoying the playground, attracting visitors year-round. Previously, it was used for kayaking and fishing, but these activities were halted due to the algae bloom. The bright green algae bloom can be clearly seen floating on the lake's surface, especially in areas with slow-moving water, as illustrated in Fig. 1(b and c) biological testing was conducted to confirm the algae species and the physical-chemical characteristics of the water.

Three sampling points were selected to represent the lake's water quality mainly focusing on areas of effluent discharge into the lake system. The sampling campaigns for each point were held in three consecutive weeks in the month of July to August 2023 presenting short dry season period data of the lake system. Approximately 1 L of grab duplicate samples were collected using polyethylene bottles. For algae count, the sample were preserved with 95 % ethanol and all the samples are stored in the cool box prior reach to laboratory.

### 2.2 Fungal cultivation

The Malaysian *Ganoderma lucidum* strain BGF4A1 was obtained from Universiti Malaya (Malaysia) and cultivated in Potato Dextrose Broth (Oxoid, UK) medium using the method described by Supramani, Jailani et al. (2019) [49]. Erlenmeyer flasks containing *G. lucidum* mycelia in the medium were incubated in a shaker incubator at 100 rpm and 26 °C for 10 days. The mycelial pellets were harvested from the growth medium by filtration using filter paper, rinsed thoroughly with distilled water to remove residual impurities, and stored in sterile distilled water at 4 °C.

### 2.3 Bio-flocculation experiment

A 1000 mL bioreactor with a working volume of 800 mL was set up in batch mode. Initially, the reactor was operated at a continuous agitation speed of 100 rpm, which was optimized for *G. lucidum* growth [47]. An inoculum volume of 1 % (w/v%) was used [37], with both adjusted (pH 4 and pH 5) and non-adjusted pH conditions. The experiment was conducted over 48 h to determine the optimal pH for the culture, with sampling intervals at 0 h (T0), 6 h (T6), 24 h (T24), 30 h (T30), and 48 h (T48). The treatment was then followed by a contact time of up to 96 h, based on the optimal pH result. The percentage of pellet volume (5 %, 10 %, and 20 %) was also evaluated. For analysis, 25 mL samples were taken from each reactor after 10 min of settling using a pipette, and the pH was measured using a pH meter (pH 2700, Eutech).

### 2.4 Analysis

The analysis is divided into two categories: lake water characterization and bio-flocculation experimentation. For the lake water characterization, the following parameters were tested: pH (pH450, Eutech), biochemical oxygen demand (BOD_5_), chemical oxygen demand (COD), ammonia–nitrogen (NH_3_–N), total phosphorus (TP), Secchi depth (SD), chlorophyll-*a* (Chl-*a*), and cyanobacteria concentration. Laboratory analysis of the water samples was conducted according to the methods outlined in the American Public Health Association (APHA) 1996 manual and standard protocols from the manufacturer (HACH, Malaysia).

Based on the lake water analysis results for Chl-*a*, TP, and SD, the eutrophication status of the study location was assessed using the Carlson Trophic State Index (CTSI) as shown in Equation (1). The lake conditions were then compared to the index values presented in Table 2 [50,51].

(1) $$\begin{array}{c} Carlson\;Trophic\;State\;Index\;\left(CTSI\right)=\frac{TSI\left(SD\right)+TSI\left(Chl-a\right)+TSI\left(TP\right)}{3}\\ \text{TSI}\left(\text{SD}\right)=60-14.41\times \ln\;\left(\text{SD}\right)\\ \begin{array}{c} \text{TSI}\left(\text{Chl}-a\right)=9.81\times \ln\left(\text{Chl}-a\right)+30.6\\ \text{TSI}\left(\text{TP}\right)=14.42\times \ln\;\left(\text{TP}\right)+4.15 \end{array} \end{array}$$

**Table 2:** Trophic state index (TSI) for eutrophication [50].

TSI	Condition	Explanation
<20	Oligotrophic	Very low algal level
20 - <40	Mesotrophic	Some algal turbidity could reduce the aesthetic appeal of the water but did not cause oxygen depletion (Condition with some algal turbidity. It could reduce aesthetic appeal in water, but not likely to cause oxygen depletion).
40 - <60	Mesotrophic	Obvious algal turbidity reduced the aesthetic appeal of the water and was likely to cause oxygen depletion.
60 - <80	Eutrophic	Condition contained high levels of algal growth, and it significantly reduced aesthetic appeal.
80 - >100	Hypereutrophic	There is a serious oxygen depletion in bottom waters

CSTI: Carlson Trophic State Index; TSI: trophic status index; Chl-*a*: chlorophyll-*a*; TP: TP: total phosphorus; SD: secchi depth.

For the bio-flocculation experiment, the efficiency of the flocculation process was evaluated using spectrophotometry by measuring the absorbance of Chl-*a* at a specific wavelength of 664 nm [52]. This measurement was based on changes in Chl-*a* concentration, as shown in Equation (2). In this equation, Abs_₀_ and Abs_t_ represent the initial and final absorbance of Chl-*a*, respectively.

(2) $$\text{Chl}-a\;\text{removal}\;\left(\%\right)=\frac{\left({\text{Abs}}_{0\;}-{\text{Abs}}_{t}\right)}{{\text{Abs}}_{o}}\times 100$$

### 2.5 Adsorption model and kinetic

The adsorption of the cyanobacterial HABs by *Ganoderma lucidum* pellets was estimated using the Chl-*a* concentration [53]. The adsorption was calculated according to Eq. (3). In this equation, q is the adsorption capacity (mg. g^−1^) of *G. lucidum* pellets at time, C_0_ and C_t_ are the concentration of Chl-*a* (mg. L^−1^) at the initial and time t, respectively; W is the weight of *G. lucidum* pellets (g), and V is the volume of solution used (L).

(3) $$q=\frac{V\left(C_{o}-C_{t}\right)}{W}$$

The Langmuir and Freundlich adsorption isotherms are used in this study and the linear equations of these isotherms are represented in Eqs. (4), (5)), respectively. Where the q_e_ (mg. g^−1^) is the amount of HABs adsorbed per unit mass of *G. lucidum* pellets at equilibrium, C_e_ (mg. L^−1^) is the equilibrium concentration of the HABs, $K_{L}$ is the Langmuir isotherm equilibrium constant (L. mg^−1^), and q_m_ is the maximum adsorption capacity (mg. g^−1^), ${\;K}_{F}$ (mg. g^−1^) and n are the Freundlich isotherm constants related to capacity and intensity [27].

(4) $$\frac{C_{e}}{q_{e}}=\frac{1}{q_{m}}C_{e}+\frac{1}{K_{L}q_{m}}$$

(5) $$\log\;{\;q}_{e}=\left(\frac{1}{n}\right)\log\;{\;C}_{e}+\log\;{\;K}_{F}\ldots \left(3\right)$$

The kinetics of HABs adsorption onto *G. lucidum* as a biosorbent were modeled using the pseudo-first order (Eq. (6)) and pseudo-second order (Eq. (7)) models. The corresponding initial adsorption rates, v_1_ and v_2_ (mg. g^−1^.min^−1^) to the two kinetic models were calculated using Eqs. (8), (9), respectively [44]. Where, q_t_ and q_e_ are the amounts HABs absorbed per biomass (mg. g^−1^) at any time and equilibrium, respectively, k_1_ is the rate constant of the first-order sorption (min^−1^), and k_2_ is the rate constant of the pseudo-second-order sorption (g. mg^−1^. min^−1^).

(6) $$\log\;\left(q_{e}-q_{t}\right)={\log\;q}_{e}-\frac{k_{1}}{0.2303}t$$

(7) $$\frac{t}{q_{t}}=\frac{1}{k_{2}q_{e}^{2}}+\frac{t}{q_{e}}$$

(8) v_1_ = k_1_q_e_

(9) v_2_ = k_2_q_e_^2^

### 2.6 Image observation

*Ganoderma lucidum* pellets were chosen and observed for changes before and after treatment. The pellets were fixed overnight at 4 °C by using 4 % glutaraldehyde (Sigma-Aldrich, USA). Subsequently, they were dehydrated by serial concentration of ethanol (30 %, 50 %, 70 %, 90 %). Finally, the pellets were dried naturally and treated with sputter-coated gold under vacuum conditions before being examined using light microscope (Euromex bScope Series Trinocular Microscope, China) and Field Emission Scanning Electron Microscopy (FESEM) Model SUPRA 55VP (CARL ZEISS, Dresden, Germany).

### 2.7 Statistical analysis

All experiments were performed in two replicate and the results present in mean ± standard deviation (s.d) using GraphPad Prism (version 9.0.0) and Microsoft Excel (2022). The significant difference (*p* < 0.05) between the tested parameters verified using Analysis of Variance (ANOVA) and advanced ANOVA test (Tukey HSD) by using IBM SPSS (version 29).

## 3 Results and discussion

### 3.1 Lake water quality

Water quality in residential lakes is crucial as it directly impacts human health and aquatic ecosystems. Poor water quality can lead to waterborne diseases, skin irritations, and the decline of aquatic biodiversity. Therefore, regular monitoring of water quality parameters is essential. Table 3 presents the physico-chemical characteristics of the lake water, revealing moderate organic loading and elevated phosphorus levels, indicating potential eutrophication. The organic content (BOD_5_ and COD) of the water were in the moderate range, with average values of 3.42 mg/L and 35.42 mg/L, respectively. However, a high concentration of TP (average of 0.615 mg/L) was identified as the main factors contributing to cyanobacterial blooms, especially when TP levels exceed 0.02 mg/L [18] The lake water exhibited an alkaline environment, where pH was measured between 9.02 and 9.51. In general, the photosynthesis process by algae species in the water removes dissolved carbon dioxide, CO_2_ (carbonic acid) leading to an increase in pH level. According to Zepernick et al. (2021), water dominated by cyanobacteria HABs typically has a pH higher than or equal to 9.2, reaching as high as pH 11. The alkaline pH range of the water provided growth advantages to the cyanobacterial due to their unique carbon concentrating mechanisms (CCMs) in an environment with high pH and low CO_2_ [54,55].

**Table 3:** Minimum (Min.), maximum (Max.), average, and standard deviation (s.d) of physico-chemical parameters measured in the Residential Park Lake in present study.

Parameters	Min.	Max.	Average ± s.d
pH	9.02	9.51	9.23 ± 0.3
Biological oxygen demand, BOD_5_	3.0 mg/L	4.0 mg/L	3.42 ± 1.0 mg/L
Chemical oxygen demand, COD	27.67 mg/L	43.67 mg/L	35.42 ± 36.9 mg/L
Ammonia nitrogen, NH_3_-N	<0.01 mg/L	<0.01 mg/L	<0.01 mg/L
Total phosphorus, TP	0.51 mg/L	0.70 mg/L	0.615 ± 0.6 mg/L
Secchi depth, SD	0.56 m	0.62 m	0.59 ± 0.3 m

**Fig. 2:**
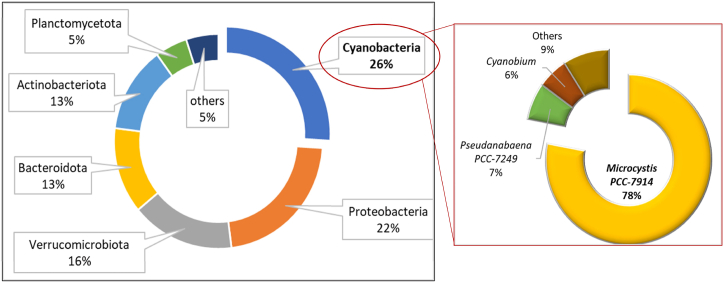
Bacterial species in lake's water dominated with Cyanobacteria genera *Microcystis* PCC-7914.

Further microbial content for the lake water was analyzed and the result as depicted in Fig. 2, showing the water mainly dominated with high proportion of cyanobacterial (26 %) and the rest of bacterial genera were proteobacteria (22 %), verrucomicrobiota (16 %), bacterioidota (13 %), actinobacteriota (13 %), planctomycetota (5 %) and others (5 %). The biological analysis also revealed the dominance of cyanobacterial species in the water, apparently, 77 % of the cyanobacterial species belong to *Microcystis* sp. (*Microcystis* PCC-7914, 77 %), while remaining consisted of *Pseudanabaena* PCC-7249 (7 %) and *Cyanobium* (6 %). The concentration of the cyanobacteria was 3400 cells/mL and the Chl-*a* content was 123 μg/L. According to Opiyo et al. (2019) [56], the assessment of the trophic state of aquatic environment is commonly assessed using TP, as it considered the most comprehensive and appropriate parameter for calculating the Carlson's trophic state index. Meanwhile, Chl-*a* is recognized as a suitable indicator for estimating the phytoplankton biomass community [57]. High levels of Chl-*a* signal deteriorate water quality, making it a valuable marker for assessing the trophic status of the aquatic ecosystems [58].

The trophic status of the lakes was calculated through the water analysis and is presented in Table 4. The results indicate that the residential park lake exhibited hypereutrophic conditions during the sampling period in August 2023. Hypereutrophy indicates that the lake is suffering from problems arising from excessive plant and algal growth due to nutrients enrichment [59].The water blooms were most likely caused by poor circulation, leading to stagnant water in many parts of the lake, combined with nutrient enrichment (especially nitrogen and phosphorus) from the drainage and runoff water from surrounding areas. The source of cyanobacterial blooms in the reservoir, as confirmed by literature, is attribute to (a) the contribution of key nutrients, primarily phosphorous (P) and nitrogen (N), including from agricultural and livestock activities; (2) discharge from residential drainages; (3) insufficient sanitation; and (4) the removal of surrounding forests [51,60,61]. Hypereutrophic due to cyanobacterial bloom outbreaks can lead to extremely high concentrations of cyanobacterial colonies and toxins, severe depletion of dissolve oxygen, and significant damage to the biodiversity of the surrounding ecosystem. The hypereutrophic urban lake leads to severe oxygen depletion, suffocate fish and other underwater organism, leading to fish kills, declining biodiversity and creating dead zones beneath the lake system [62]. The hypereutrophic creating dead zone, unpleasant odor, habitat loss and health implication the human, pets and wild life thus require urgent mitigation to address the issue. Thus, current work discusses alternative solutions to address the issue by using *Ganoderma lucidum* as discussed in following section.

**Table 4:** Carlson's Trophic State Index of the residential park lake.

TSI(SD)	TSI(Chl-*a*)	TSI(TP)	CSTI	Trophic Status
68.355	77.808	96.750	80.971	Hypereutrophic

### 3.2 Bio-flocculation efficiency of *Ganoderma lucidum*

**Fig. 3:**
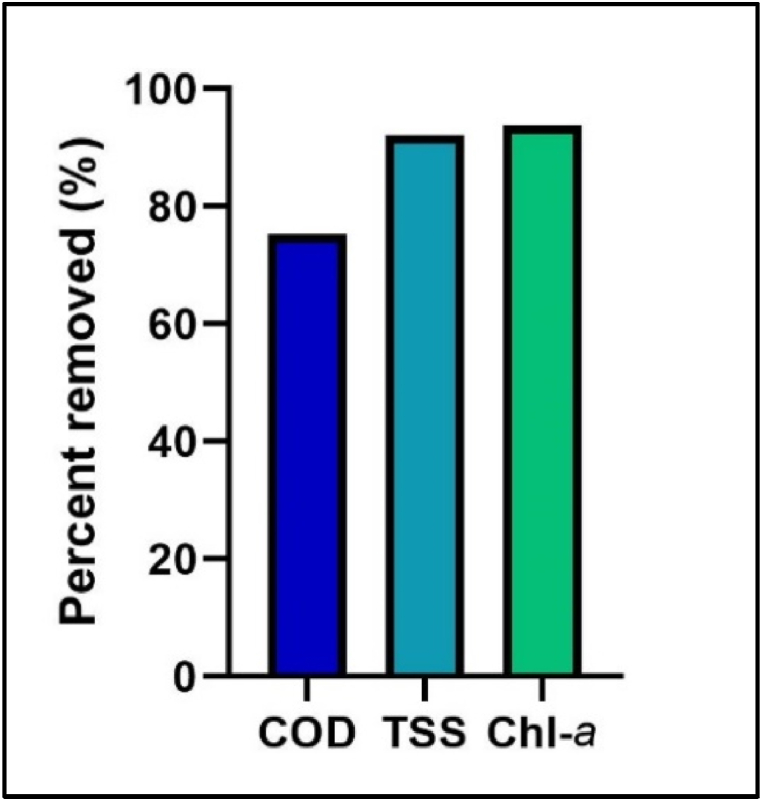
The effectiveness of *Ganoderma lucidum* under optimized conditions (1 % inoculum concentration, 48-h of treatment time, pH 4100 rpm agitation speed).

Field observations indicate that the residential park lake is experiencing severe eutrophication due to excessive nutrient input, particularly phosphorus, and poor water circulation. To address this issue, this study investigates the use of *Ganoderma lucidum* as a bio-flocculant to improve water quality. The results demonstrate that the bio-flocculation process effectively removed approximately 75.2 % of the COD, flocculate*d* 92.09 % of the total suspended solids (including the algae), and removed 93.70 % of Chl-*a* under optimized conditions, as illustrated in Fig. 3. The effects of each test parameter are discussed in the following section.

#### 3.2.1 Effect of pH

**Fig. 4:**
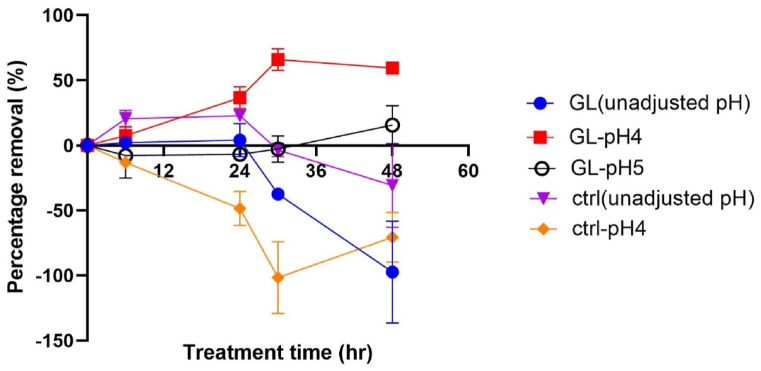
Comparison of effect of uncontrolled pH and pH 4 (condition: 1 % inoculum, 100 rpm, room temperature) (Table S1).

The optimal pH range can significantly influence the performance of fungal treatments, particularly for *Ganoderma lucidum*, which generally thrives in slightly acidic conditions between pH 4 and 5 [37,63,64]. Therefore, this study focused on a pH range of 4–5 to evaluate the bio-flocculation efficiency of *Ganoderma lucidum*. The results are presented in Fig. 4. The results demonstrated that within 48 h of treatment, *G. lucidum* pellets effectively flocculated cyanobacteria at pH 4 compared to the pH 5, the unadjusted pH (pH 8.57) and the control reactor. The highest flocculation efficiency achieved was 72 % after 30 h of treatment, while the reactor with unadjusted pH did not show any significant decrease after 24 h of treatment. The pH 5 treatment showed a slight increase in removal efficiency, reaching 26 % after 48 h of bio-flocculation. A one-way ANOVA confirmed that the differences in removal performance across different pH levels were statistically significant. (*p* < 0.05) (Fig. S1).

pH can alter the surface properties of the pellets and change cellular metabolic pathways by affecting the degree of decomposition and ionization of nutrients in the water [18]. In a previous research, *G. lucidum* efficiently removed COD and NH₃-N at the optimal pH of 4 [63], while another type of fungus, *Aspergillus* sp., efficiently co-pelletized microalgae in an acidic environment with a pH range of 4.67 [65]. Moreover, according to previous studies, algae generally possess a negative charge on their surface due to the presence of carboxylic (-COOH), amino (-NH₂), and phosphate (-PO₄) groups [66], making them stable suspensions in alkaline pH (pH > 9.0). On the other hand, fungal pellets consist of positively charged surfaces [67], acting as cationic cells. They are easily acclimatizing to acidic environments while attracting negatively charged algae.

#### 3.2.2 Effect of contact time

**Fig. 5:**
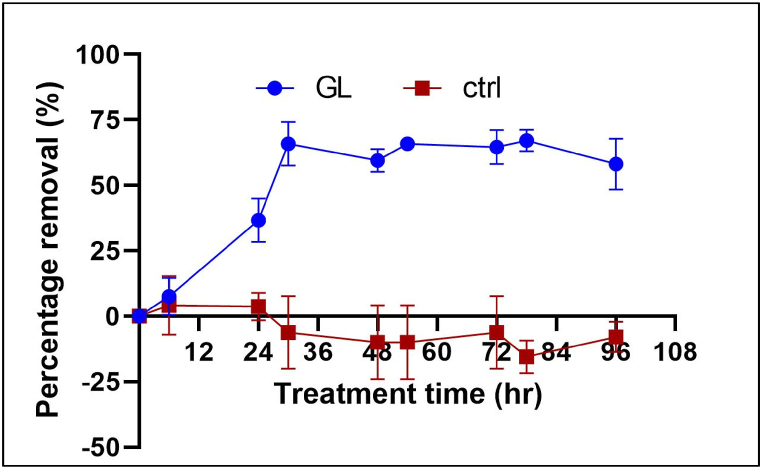
Effect of treatment time (condition: pH 4, 1 % inoculum volume),100 rpm, room temperature) (Table S2).

The flocculation time at room temperature (∼25 °C) with initial pH adjustment (pH 4) is presented in Fig. 5. Fungal pellets showed sustained adsorption of the cyanobacteria with increasing flocculation time, remaining constant up to 96 h of treatment time. The results demonstrate that the algae achieved maximum flocculation efficiency at 30 and 48 h, which are ideal time points for further optimization experiments to ensure maximum adsorption. In previous studies, the effective time for algae flocculation using fungal treatment has varied from 3-4 h to 4 days of contact time. Most of these studies focused on flocculating *Chlorella* sp. using fungi like *Aspergillus* sp., which efficiently flocculated algae up to 99 % within 3–4 h at 100 rpm and 38 °C [67]. However, in a similar study, Xie et al. (2013) used *Cunninghamella echinulate*, another fungal species, and achieved the highest flocculation (99 %) at 48 h of contact time.

#### 3.2.3 Effect of dosage volume

**Fig. 6:**
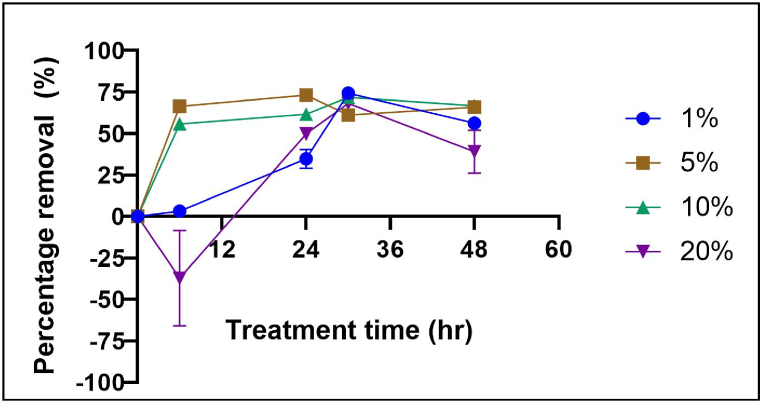
Effect of inoculum volume at condition of initial pH 4, 100 rpm agitation and room temperature (Table S3).

To explore the contribution of the amount of fungal biomass to the constant algae concentration (at approximately *Abs*_*0*_ = 0.05), different pellet concentrations (1 %, 5 %, 10 % and 20 %, w/v) were used for a 48 h treatment time. The results demonstrate that 1 % of *G. lucidum* inoculum is sufficient to achieve high adsorption and is comparable to higher volumes of 5 %, 10 % and 20 %, as shown in Fig. 6. Increasing the inoculum volume did not improve the treatment time or the percentage efficiency for the current algae concentration. A one-way ANOVA revealed that there was no significant difference (*p* > 0.05) in algal removal efficiency among different inoculum volumes, as shown in Fig. S2. Excessive fungal biomass in the reactor can lead to an increase in repulsive charge, hindering the attachment of algae cells to the biomass surface. A previous study by Nie et al. [27], used fungi (*Aspergillus oryzae*) at an optimal volume of 11.0 g/L (1.1 %) to flocculate more than 80 % of *Microcystis aeruginosa* within 4 days. However, a linear relationship between flocculation efficiency and algae concentration is expected, with higher algae concentrations corresponding to higher available surface areas of the adsorbent.

#### 3.2.4 Cyanobacterial removal

**Fig. 7:**
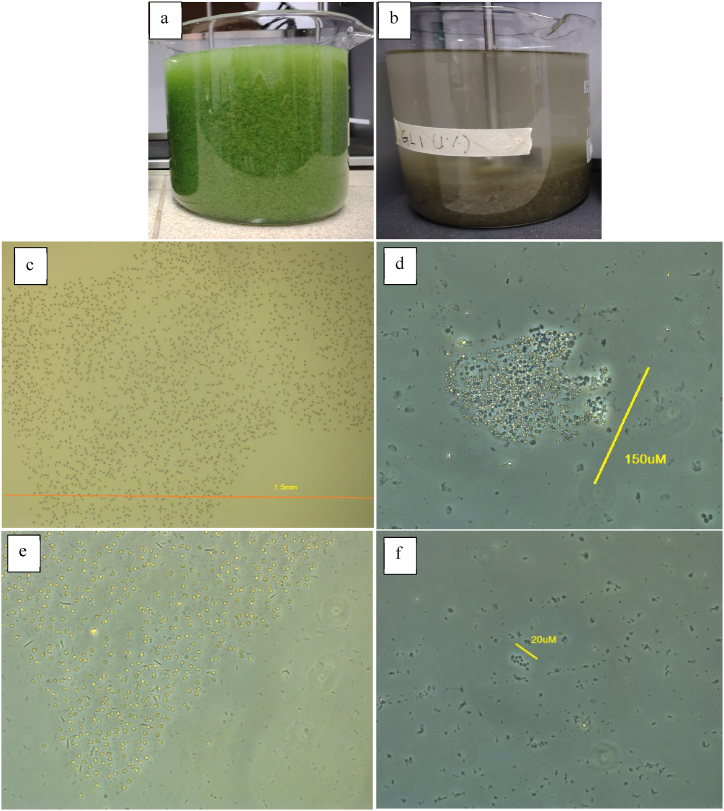
The images of the cyanobacteria show (a) before treatment: green and suspended; (b) after treatment: settled at the bottom and brownish in color; under light microscope at 100× magnification; (c, e) *Microcystis* colonies before treatment were observed to have sizes ranging from 3 to 10 mm; (d, f) *Microcystis* colonies after treatment were observed to have sizes less than 200 μm.

Following treatment, most cyanobacteria clumped together and sank to the reactor's bottom (Fig. 7-b), unlike the untreated sample (Fig. 7-a) which displayed suspended, bright green algae. The treated water and the untreated samples were examined under a microscope for cyanobacterial counts, focusing primarily on *Microcystis* colonies count, and the results are shown in Fig. 7(c–f). Before treatment, the samples contained large and intact *Microcystis* large colonies ranging from 3 mm to 10 mm, with a count of 2700/mL, while after treatment, the is a significant difference of the large colonies' breakdown into smaller colonies size range less than 200 μm, where estimated number was 8850/mL. The after-treatment colonies count higher than before treatment is resulting from the breakdown of from the initial large colonies into smaller colonies and it does not show the signed of multiplying new colonies. Furthermore, the observation of the *Microcystis* after treatment showed that the colonies dyeing as the color changing from bright green to pale green color as shown in Fig. 6b showing the algicide properties of the fungi. This finding is consistent with Wang et al. (2022), who reported that fungal treatment can lead to algal cell lysis and sedimentation. *G. lucidum* likely damages algal cells directly and secretes enzymes that contribute to cell breakdown and colony disintegration.

As the results indicate, most of the *Microcystis* colonies were breaking down under experimental conditions, with few intact, smaller colonies remaining, suggesting that the cyanobacteria were already dead or dying. Aside from bio-flocculation, colony deformation has also been reported as a strategy to reduce the damage caused by cyanobacterial colonies, including continuous attacks by fungi that inhibit the normal physiological and metabolic functions of HAB cells [41,68]. Smaller colonies, which possess thinner floating colonies covering the surface layers, can improve light penetration into the water below. Conversely, large colonies, which contain a high content of extracellular polymeric substances (EPS), can interfere with the fungi's effectiveness by protecting the cells from adverse environmental effects [69].

### 3.3 Adsorption isotherm and kinetics

Adsorption isotherms are important for studying the surface properties and affinities of *G. lucidum* as a biosorbent and the degree of cyanobacterial adsorption. For the adsorption study, cyanobacterial cells from the optimal reactors were selected to represent the isotherm and kinetic models.

#### 3.3.1 Adsorption isotherm

The data obtained from the linear isotherms is shown in Table 5, which indicates that both the Langmuir and Freundlich models satisfy the assumptions for the algal cells and *G. lucidum* biomass models.

**Table 5:** Langmuir and Freundlich isotherm constants for the adsorption of cyanobacterial cells onto *Ganoderma lucidum* pellets. (Experimental conditions: absorbent dosage = 10.0 g per 1000 mL, mixing rate = 100 rpm, T = 25 ± 1 °C).

Langmuir isotherm				Freundlich isotherm			
q_m_ (mg/g)	K_L_ (L/mg)	R_L_	R^2^	K_F_ (mg/g)	n	1/|n|	R^2^
6.3131	−0.0055	0.2659	0.9868	80130.9	−0.6733	1.4852	0.9883

Both models exhibited equivalently high R^2^ values of 0.9868 and 0.9883, respectively, suggesting that monolayer and multilayer adsorption occurred simultaneously during the treatment process [27]. Based on the Langmuir equilibrium constant value, K_L_, the adsorption efficiency can be predicted using the constant parameter, R_L_ = 1/(1+K_L_C_o_) [70]. The calculated R_L_ value of 0.2659 falls between 0 and 1, indicating favorable and homogeneous adsorption of algal cells into the biomass. Meanwhile, values of R_L_ > 1, R_L_ = 1, or R_L_ = 0 indicate unfavorable, linear, and irreversible adsorption processes, respectively. On the other hand, the maximum adsorption capacity of the *G. lucidum* biomass is 6.3131 mg/g.

For the Freundlich model, the value of 1/n can be used to predict the intensity of cyanobacterial adsorption onto *G. lucidum*, where a value between 0 and 1 indicates a favorable isotherm, and a value greater than 1 indicates an unfavorable adsorption. The obtained value of 1/n greater than 1 suggests that cyanobacterial adsorption onto *G. lucidum* is unfavorable and heterogeneous. This result indicates that cyanobacterial cells are more favorably and homogeneously adsorbed on the surface of *G. lucidum* pellets. The results are comparable to those of [27], where *Microcystis* were potentially homogenously distributed on *A. oryzae* cells as adsorption sites.

#### 3.3.2 Adsorption kinetics

Two kinetic models (pseudo-first order and pseudo-second-order) were used to evaluate the adsorption performance based on the reaction rate and adsorption quantity. The pseudo-first order model, also known as Lagergren's model, is primarily used to describe physical adsorption (physisorption) in solid–liquid systems [71], while the pseudo-second-order rate expression is applied to analyze chemical adsorption (chemisorption) from liquid solutions [72]. The relevant parameters associate with these models are presented in Table 6. The pseudo-second order model was found to explain cyanobacterial adsorption most effectively, as evidenced by its higher correlation, R^2^ value of 0.9761 compared to the pseudo-first order of 0.7655. The pseudo-order model also showed a calculated adsorption equilibrium value, q_e_ (18.975 mg/g), that was close to the experimental data (21.435 mg/g). Thus, experimental data was best described using pseudo-second order model, with an adsorption rate, v_2_ of 0.01035 mg/g/min. The results indicate that the adsorption process was primarily controlled by chemisorption, involving chemical bonds and surface functional groups.

**Table 6:** Kinetic pseudo-first order and pseudo-second order parameters of cyanobacterial cells onto *Ganoderma lucidum* pellets. (Experimental conditions: absorbent dosage = 10.0 g per 1000 mL, mixing rate = 100 rpm, T = 25 ± 1 °C).

**Pseudo-first order (1st order)**	k_1_(1/min)	0.0102	
	q_e_ exp (mg/g)	21.435	
	q_e_ (mg/g)	16.577	
	v_1_ (mg/g.min)	0.0389	
	R^2^	0.7655	
**Pseudo-second order (2nd order)**	k_2_(g/mg.min)	0.000545	
	q_e_ exp (mg/g)	21.435	
	q_e_ (mg/g)	18.975	
	v_2_ (mg/g.min)	0.01035	
	R^2^	0.9761	

The adsorption study also aimed to determine the adsorption mechanism based on the contact time compared to the pseudo-first order and pseudo-second order models, as shown in the attached plot in Table 6. It was observed that the adsorption for cyanobacterial cells increased rapidly during the first 30 h of contact time. After 30 h, the rate decreased until a constant adsorption capacity was reached through 96 h of treatment. The contact time of 48 h was assumed to represent the equilibrium time at which adsorption equilibrium occurred. Although the initial findings primarily supported the pseudo-second order model over the pseudo-first order model, the kinetic curves showed a contrary trend, with the pseudo-first order model being more relevant to the actual adsorption process. However, the curve for the pseudo-second order model still followed a similar pattern to both the actual model and the pseudo-first order kinetic model.

### 3.4 Image analysis

**Fig. 8:**
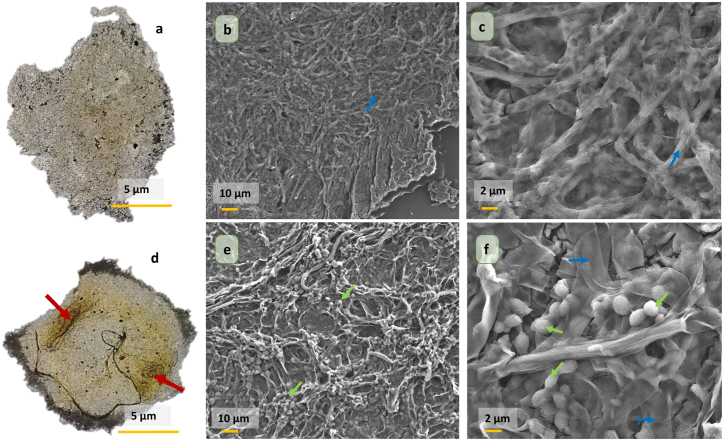
Macroscopic observation (Light Microscope Magnification 4X-left pictures) and microscopic observation (Field Emission Scanning Electron Microscopy-middle pictures: Magnification 500X and right pictures: Magnification 2000X) of *Ganoderma lucidum* pellets; a, b and c before treatment; d, e and f after treatment at optimized parameters (1 %, 48 h, pH4, 100 rpm); red arrows indicate algae adsorption on biomass; blue arrows indicate the hyphae of the fungi and green arrows indicate the adsorption of the cyanobacterial cells.

To understand the mechanism of cyanobacterial adsorption onto *G. lucidum* pellets, samples were evaluated before and after treatment under macroscopic and microscopic observation. Fig. 8 shows a comparison of the untreated *G. lucidum* pellets (before treatment) and the treated pellets (after treatment) under a light microscope (LM) and Field Emission Scanning Electron Microscopy (FESEM). Under LM, the untreated pellets (image a) exhibited a consistent grayish color on the surface, while after treatment (image d), the red arrow showed the pellets changed to a deep yellowish-green color, indicating the adsorption of cyanobacterial cells onto the fungal mycelia. Moreover, FESEM observations revealed that a large number of cyanobacterial cells had attached and were trapped on the fungal hyphae, as indicated by the green arrows in Fig. 8(e and f). This implies that the cells were successfully adsorbed by the fungi. The pellets remained intact throughout the flocculation treatment process, demonstrating their stability and preventing the detachment of cyanobacterial cells. Furthermore, the structure of the cyanobacterial cells remained intact within the hyphae, thus reducing the release of extracellular secretions and further colony formation by the harmful algae [27].

## 4 Conclusions

Harmful cyanobacterial blooms have been one of the most challenging ecological problems faced by freshwater ecosystems. The residential park lake, a popular community hub for recreational activities, also faces the problem. The evaluation of the lake's eutrophication status using the Carlson Trophic State Index (CTSI) was 80.97, indicating that the lake exhibits hypereutrophic conditions during the sampling period. The hypereutrophic conditions lead to severe oxygen depletion in the system and loss of biodiversity. The physico-chemical analysis of the lake water quality showed that the water was moderately rich with organic content, with an average COD of 35.42 mg/L with excessive concentration of phosphorus. Poor water circulation system is also a factor contributing to the hypereutrophic status of the lake. Microbiological analysis confirms the dominance of Microcystis PCC-7914 colonies within the cyanobacterial harmful algal blooms (HABs), which can produce significant toxins in the water.

As a mitigation approach, this study utilizes a biological approach to bio-flocculate the cyanobacterial biomass using *Ganoderma lucidum* strain BGF4A1 pellets. The experimental results demonstrate that *G. lucidum* efficiently bio-flocculated the cyanobacterial biomass (as measured by Chl-a Abs664 nm readings) up to 93.70 % under conditions of pH 4, 100 rpm agitation, and 1 % inoculum mass. Furthermore, approximately 75.28 % of chemical oxygen demand (COD) was reduced, and 92.09 % of total suspended solids (TSS) were flocculated from the water. Image analysis confirmed that some bacterial cells were deposited on the hyphae of the fungi, indicating the pellets’ promising ability to adsorb the cells within 48 h of contact time. The cyanobacterial count also showed that the cells lost their ability to clump as colonies and were dying as individual cells.

Using *Ganoderma lucidum* provides promising potential to be applied as the bio-flocculation agent in remediating harmful algal blooms. Certain limitations have been identified through the lab-scale experimental work, where the treatment is pH sensitive, as knowing it will be effective once the environment is slightly acidic. Scaling up the Ganoderma-technology wastewater treatment systems needs to be explored more in the future due to factors such as nutrient requirements, oxygen supply, and waste disposal. Despite these limitations, *Ganoderma lucidum* remains a promising bioremediation agent with potential for further development and optimization. By addressing these challenges and combining it with other treatment technologies, it may be possible to create more efficient and sustainable wastewater treatment systems. Apart from that, more attention should be paid to the effects of nutrients in the lake, and measures to maintain good water flow should be taken for the sustainable restoration of the aquatic ecosystem.

## CRediT authorship contribution statement

**Zarimah Mohd Hanafiah:** Writing – original draft, Methodology, Conceptualization. **Anggita Rahmi Hafsari:** Methodology, Formal analysis. **Malini Elango:** Writing – original draft, Methodology, Formal analysis. **Zul Ilham:** Methodology, Conceptualization. **Febri Doni:** Writing – review & editing. **Wan Hanna Melini Wan Mohtar:** Writing – review & editing. **Yusufjon Gafforov:** Resources, Data curation. **Yong Jie Wong:** Resources, Data curation. **Khairul Nizam Abdul Maulud:** Writing – review & editing. **Nor Hidayah Ismail:** Writing – review & editing. **Mohd Yusmiaidil Putera Mohd Yusof:** Funding acquisition. **Wan Abd Al Qadr Imad Wan-Mohtar:** Supervision, Resources, Funding acquisition, Conceptualization.

## Data availability statement

Not additional data was used for the research described in the article.

## Funding

This research was funded by Water Quality Performance Of TMK, Kuala Lumpur: Phase 2 Public Awareness and BioDeF System WBS Account UM.0000183/HRU.RK Universiti Malaya. Project No. MG003-2024, PV085-2024: Performance Of Panasonic Water Filtration Alkaline Ionizer On Acidic Food And Beverages (Panasonic Malaysia Sdn Bhd) and IF084-2024: Sampling And Isolation Of Tropical Thraustochytrids From Malaysian Mangroves (Brudy Technology, Spain)

## Declaration of competing interest

The authors declare the following financial interests/personal relationships which may be considered as potential competing interests:Wan Abd Al Qadr Imad Wan-Mohtar reports financial support was provided by 10.13039/501100004386University of Malaya. If there are other authors, they declare that they have no known competing financial interests or personal relationships that could have appeared to influence the work reported in this paper.

## References

[1] Kataoka T., Ohbayashi K., Kobayashi Y., Takasu H., Nakano S.I., Kondo R., Hodoki Y. (2020). Distribution of the harmful bloom-forming cyanobacterium, microcystis aeruginosa, in 88 freshwater environments across Japan. Microb. Environ..

[2] Grogan A.E., Alves-de-Souza C., Cahoon L.B., Mallin M.A. (2023). Harmful algal blooms: a prolific issue in urban stormwater ponds. Water (Switzerland).

[3] Dziga D., Maksylewicz A., Maroszek M., Budzyńska A., Napiorkowska-Krzebietke A., Toporowska M., Grabowska M., Kozak A., Rosińska J., Meriluoto J. (2017). The biodegradation of microcystins in temperate freshwater bodies with previous cyanobacterial history. Ecotoxicol. Environ. Saf..

[4] Sellner K.G., Doucette G.J., Kirkpatrick G.J. (2003). Harmful algal blooms: causes, impacts and detection. J. Ind. Microbiol. Biotechnol..

[5] Karlson B., Andersen P., Arneborg L., Cembella A., Eikrem W., John U., West J.J., Klemm K., Kobos J., Lehtinen S., Lundholm N., Mazur-Marzec H., Naustvoll L., Poelman M., Provoost P., De Rijcke M., Suikkanen S. (2021). Harmful algal blooms and their effects in coastal seas of Northern Europe. Harmful Algae.

[6] Rastogi R.P., Madamwar D., Incharoensakdi A. (2015). Bloom dynamics of cyanobacteria and their toxins: environmental health impacts and mitigation strategies. Front. Microbiol..

[7] Kubickova B., Babica P., Hilscherová K., Šindlerová L. (2019). Effects of cyanobacterial toxins on the human gastrointestinal tract and the mucosal innate immune system. Environ. Sci. Eur..

[8] Igwaran A., Kayode A.J., Moloantoa K.M., Khetsha Z.P., Unuofin J.O. (2024). Cyanobacteria harmful algae blooms: causes, impacts, and risk management. Water Air Soil Pollut..

[9] Svirčev Z., Chen L., Sántha K., Drobac Backović D., Šušak S., Vulin A., Palanački Malešević T., Codd G.A., Meriluoto J. (2022). A review and assessment of cyanobacterial toxins as cardiovascular health hazards. Arch. Toxicol..

[10] Alosman M., Cao L., Massey I.Y., Yang F. (2021). The lethal effects and determinants of microcystin-LR on heart: a mini review. Toxin Rev..

[11] Cao L., Massey I.Y., Feng H., Yang F. (2019). A review of cardiovascular toxicity of microcystins. Toxins.

[12] Sukenik A., Kaplan A. (2021). Cyanobacterial harmful algal blooms in aquatic ecosystems: a comprehensive outlook on current and emerging mitigation and control approaches. Microorganisms.

[13] Yu H., Lei P., Ma J., Jin J., Ma Y., Fang Y., Zeng G., Zhang K., Jin L., Sun D. (2023). The potential of white-rot fungi for algal control: mechanisms, Strategies, and Challenges. Environ. Res..

[14] Rodríguez E., Onstad G.D., Kull T.P.J., Metcalf J.S., Acero J.L., von Gunten U. (2007). Oxidative elimination of cyanotoxins: comparison of ozone, chlorine, chlorine dioxide and permanganate. Water Res..

[15] Song J., Xu Z., Chen Y., Guo J. (2023). Nanoparticles, an emerging control method for harmful algal blooms: current technologies, challenges, and perspectives. Nanomaterials.

[16] Xia Z., Yuan H., Liu J., Sun Y., Tong Y., Zhao S., Xia J., Li S., Hu M., Cao J., Zhang J., He P. (2022). A review of physical, chemical, and biological green tide prevention methods in the Southern Yellow Sea. Mar. Pollut. Bull..

[17] Zeng G., Wang P., Wang Y. (2015). Algicidal efficiency and mechanism of Phanerochaete chrysosporium against harmful algal bloom species. Algal Res..

[18] Zeng G., Zhang R., Liang D., Wang F., Han Y., Luo Y., Gao P., Wang Q., Wang Q., Yu C., Jin L., Sun D. (2023). Comparison of the advantages and disadvantages of algae removal technology and its development status. Water (Switzerland).

[19] Huang H., Wu G., Sheng C., Wu J., Li D., Wang H. (2020). Improved cyanobacteria removal from harmful algae blooms by two-cycle, low-frequency, low-density, and short-duration ultrasonic radiation. Water (Switzerland).

[20] Huang W., Chu H., Dong B., Hu M., Yu Y. (2015). A membrane combined process to cope with algae blooms in water. Desalination.

[21] Wang B., Song Q., Long J., Song G., Mi W., Bi Y. (2019). Optimization method for Microcystis bloom mitigation by hydrogen peroxide and its stimulative effects on growth of chlorophytes. Chemosphere.

[22] Plummer J.D., Edzwald J.K. (2002). Effects of chlorine and ozone on algal cell properties and removal of algae by coagulation. J. Water Supply Res. Technol. - Aqua.

[23] Hu L., Yang Z., Pan X., Zhao N., Peng J., Wan C. (2017). Use of fish species from different trophic levels to control algae and water quality: an enclosure experiment in eutrophic area of Xiaojiang River. PLoS One.

[24] Sinang S.C., Daud N., Kamaruddin N., Poh K.B. (2019). Potential growth inhibition of freshwater algae by herbaceous plant extracts. Acta Ecol. Sin..

[25] Hu J., Kokoette E., Xu C., Huang S., Tang T., Zhang Y., Liu M., Huang Y., Yu S., Zhu J., Holmer M., Xiao X. (2023). Natural algaecide sphingosines identified in hybrid straw decomposition driven by white-rot fungi. Adv. Sci..

[26] Muradov N., Taha M., Miranda A.F., Wrede D., Kadali K., Gujar A., Stevenson T., Ball A.S., Mouradov A. (2015). Fungal-assisted algal flocculation: application in wastewater treatment and biofuel production. Biotechnol. Biofuels.

[27] Nie Y., Wang Z., Wang W., Zhou Z., Kong Y., Ma J. (2022). Bio-flocculation of Microcystis aeruginosa by using fungal pellets of Aspergillus oryzae: performance and mechanism. J. Hazard Mater..

[28] Thongdam S., Kuster A.C., Huser B.J., Kuster A.T. (2021). Low dose coagulant and local soil ballast effectively remove cyanobacteria (Microcystis) from tropical lake water without cell damage. Water (Switzerland).

[29] Xie S., Sun S., Dai S.Y., Yuan J.S. (2013). Efficient coagulation of microalgae in cultures with filamentous fungi. Algal Res..

[30] Li H., Wang J., Zhang E., Shao Y., Yang L., Yang B., Tan Y., Gao T. (2022). Cumulative effects of physical. Chemical, and Biological Measures on Algae Growth Inhibition, Water (Switzerland).

[31] Aba R.P., Sbahi S., Mugani R., Redouane E.M., Hejjaj A., Azevedo J., Moreira C.I.T., Boo S.F., Alexandrino D.A.D.M., Campos A., Vasconcelos V., Oudra B., Ouazzani N., Mandi L. (2024). Eco-friendly management of harmful cyanobacterial blooms in eutrophic lakes through vertical flow multi-soil-layering technology. J. Hazard Mater..

[32] Mugani R., El Khalloufi F., Aba R.P., Redouane E.M., Haida M., Essadki Y., Zerrifi S.E.A., Hejjaj A., Ouazzani N., Azevedo J., Campos A., Grossart H.P., Vasconcelos V., Oudra B., Mandi L. (2024). Innovative approaches for Microcystin removal: bacterioplankton biodegradation and multi-soil-layering system performance assessment. J. Clean. Prod..

[33] Bavithra G., Azevedo J., Oliveira F., Morais J., Pinto E., Ferreira I.M.P.L.V.O., Vasconcelos V., Campos A., Almeida C.M.R. (2020). Assessment of constructedwetlands’ potential for the removal of cyanobacteria and microcystins. (MC-LR), Water (Switzerland).

[34] Nwankwegu A.S., Li Y., Huang Y., Wei J., Norgbey E., Sarpong L., Lai Q., Wang K. (2019). Harmful algal blooms under changing climate and constantly increasing anthropogenic actions: the review of management implications. 3 Biotech.

[35] Anabtawi H.M., Lee W.H., Al-Anazi A., Mohamed M.M., Aly Hassan A. (2024). Advancements in biological strategies for controlling harmful algal blooms. HABs), Water (Switzerland).

[36] Usmani Z., Sharma M., Lukk T., Gupta V.K. (2021). Role of Fungi in Bioremediation of Soil Contaminated with Persistent Organic Compounds.

[37] Hanafiah Z.M., Wan Mohtar W.H.M., Hasan H.A., Jensen H.S., Klaus A., Sharil S., Wan-Mohtar W.A.A.Q.I. (2022). Ability of Ganoderma lucidum mycelial pellets to remove ammonia and organic matter from domestic wastewater. Int. J. Environ. Sci. Technol..

[38] Mohd Hanafiah Z., Wan Mohtar W.H.M., Wan-Mohtar W.A.A.Q.I., Bithi A.S., Rohani R., Indarto A., Yaseen Z.M., Sharil S., Binti Abdul Manan T.S. (2024). Removal of pharmaceutical compounds and toxicology study in wastewater using Malaysian fungal Ganoderma lucidum. Chemosphere.

[39] Mohd Hanafiah Z., Radzi Azmi A., Abd Al Qadr Imad Wan-Mohtar W., Olivito F., Golemme G., Ilham Z., Ainurzaman Jamaludin A., Razali N., Abdul Halim-Lim S., Hanna Melini Wan Mohtar W., Land G., Gamuda M. (2024). Water quality assessment and decolourisation of contaminated Ex-mining lake water using bioreactor dye-eating fungus (BioDeF) system: a real case study. Toxics.

[40] Wan Mohtar W.H.M., Wan-Mohtar W.A.A.Q.I., Zahuri A.A., Ibrahim M.F., Show P.L., Ilham Z., Jamaludin A.A., Abdul Patah M.F., Ahmad Usuldin S.R., Rowan N. (2022). Role of ascomycete and basidiomycete fungi in meeting established and emerging sustainability opportunities: a review. Bioengineered.

[41] Wang J., Zeng G., Wang F., Huang X., Li Y., Liang D., Zhang M., Sun D. (2022). Study on the algae lysis method of white rot fungi algae control system. Water (Switzerland).

[42] Chang J., Zhang H., Cheng H., Yan Y., Chang M., Cao Y., Huang F., Zhang G., Yan M. (2020). Spent Ganoderma lucidum substrate derived biochar as a new bio-adsorbent for Pb2+/Cd2+ removal in water. Chemosphere.

[43] Mooralitharan S., Hanafiah Z.M., Manan T.S.B.A., Hasan H.A., Jensen H.S., Wan-Mohtar W.A.A.Q.I., Mohtar W.H.M.W. (2021). Optimization of mycoremediation treatment for the chemical oxygen demand (COD) and ammonia nitrogen (AN) removal from domestic effluent using wild-Serbian Ganoderma lucidum (WSGL). Environ. Sci. Pollut. Res. Int..

[44] Zahuri A.A., Wan Mohtar W.H.M., Hanafiah Z.M., Abdul Patah M.F., Show P.L., Gafforov Y., Wan-Mohtar W.A.A.Q.I. (2024). Mycoremediation of industrial textile wastewater using Ganoderma lucidum pellets and activated dolomite in batch bioreactor. Mol. Biotechnol..

[45] Zhang J., Li Y., Shao E., Chow V., Li J., Qian J., Xu P., Li J., Song H., Zhou W., Shao S. (2023). Feasibility and constraints of edible fungi bio-flocculating microalgae. Algal Res..

[46] Gong X., Wang Y., Huang D., Zhang J. (2022). Effects of microplastics of different sizes on the Chlorella vulgaris - Ganoderma lucidum co-pellets formation processes. Sci. Total Environ..

[47] Supramani S., Ahmad R., Ilham Z., Suffian Mohamad Annuar M., Abd Al Qadr Imad Wan-Mohtar W., Klaus A. (2019). Optimisation of biomass, exopolysaccharide and intracellular polysaccharide production from the mycelium of an identified Ganoderma lucidum strain QRS 5120 using response surface methodology. AIMS Microbiol.

[48] Wan-Mohtar W.A.A.Q.I., Taufek N.M., Thiran J.P., Rahman J.F.P., Yerima G., Subramaniam K., Rowan N. (2021). Investigations on the use of exopolysaccharide derived from mycelial extract of Ganoderma lucidum as functional feed ingredient for aquaculture-farmed red hybrid Tilapia (Oreochromis sp.). Future Foods.

[49] Supramani S., Jailani N., Ramarao K., Mohd Zain N.A., Klaus A., Ahmad R., Wan-Mohtar W.A.A.Q.I. (2019). Pellet diameter and morphology of European Ganoderma pfeifferi in a repeated-batch fermentation for exopolysaccharide production. Biocatal. Agric. Biotechnol..

[50] Carlson R.E. (1977). A trophic state index for lakes. Limnol. Oceanogr..

[51] Lin J.L., Karangan A., Huang Y.M., Kang S.F. (2022). Eutrophication factor analysis using Carlson trophic state index (CTSI) towards non-algal impact reservoirs in Taiwan. Sustainable Environment Research.

[52] Dunne R.P. (1999).

[53] U. Epa, E. Response Team, STANDARD OPERATING PROCEDURES CHLOROPHYLL DETERMINATION CONTENTS, n.d.

[54] Zepernick B.N., Gann E.R., Martin R.M., Pound H.L., Krausfeldt L.E., Chaffin J.D., Wilhelm S.W. (2021). Elevated pH conditions associated with microcystis spp. blooms decrease viability of the cultured diatom fragilaria crotonensis and natural diatoms in lake erie. Front. Microbiol..

[55] Sandrini G., Tann R.P., Schuurmans J.M., van Beusekom S.A.M., Matthijs H.C.P., Huisman J. (2016). Diel variation in gene expression of the CO2-concentrating mechanism during a harmful cyanobacterial bloom. Front. Microbiol..

[56] Opiyo S., Sitoki L., Ogendi G.M., Balaka Opiyo S., Mochache Getabu A., Morara Sitoki L., Shitandi A., Mokua Ogendi G., Stephen Balaka Opiyo C. (2019). Application of the Carlson's trophic state index for the assessment of trophic status of lake simbi ecosystem, a deep alkaline-saline Lake in Kenya. Int J Fish Aquat Stud.

[57] García–Nieto P.J., García–Gonzalo E., Alonso Fernández J.R., Díaz Muñiz C. (2024). Forecast of chlorophyll-a concentration as an indicator of phytoplankton biomass in El Val reservoir by utilizing various machine learning techniques: a case study in Ebro river basin, Spain. J. Hydrol. (Amst.).

[58] Guo J., Lu J., Zhang Y., Zhou C., Zhang S., Wang D., Lv X. (2022). Variability of chlorophyll-a and secchi disk depth (1997–2019) in the bohai sea based on monthly cloud-free satellite data reconstructions. Rem. Sens..

[59] Husk B., Julian P., Simon D., Tromas N., Phan D., Painter K., Baulch H., Sauvé S. (2024). Improving water quality in a hypereutrophic lake and tributary through agricultural nutrient mitigation: a Multi-year monitoring analysis. J. Environ. Manag..

[60] Viso-Vázquez M., Acuña-Alonso C., Rodríguez J.L., Álvarez X. (2021). Remote detection of cyanobacterial blooms and chlorophyll-a analysis in a eutrophic reservoir using sentinel-2. Sustainability.

[61] Azman M.H.A., Hamdan R., Ali Z.M., Siddiqui Z. (2023). Water quality monitoring for trophic state of tasik kemajuan, Universiti tun hussein onn Malaysia. International Journal of Sustainable Construction Engineering and Technology.

[62] Norris B., Laws E.A. (2017). Nutrients and phytoplankton in a shallow, hypereutrophic urban lake: prospects for restoration. Water (Switzerland).

[63] Mohd Hanafiah Z., Wan Mohtar W.H.M., Abu Hasan H., Jensen H.S., Klaus A., Wan-Mohtar W.A.A.Q.I. (2019). Performance of wild-Serbian Ganoderma lucidum mycelium in treating synthetic sewage loading using batch bioreactor. Sci. Rep..

[64] Mir-Tutusaus J.A., Baccar R., Caminal G., Sarrà M. (2018). Can white-rot fungi be a real wastewater treatment alternative for organic micropollutants removal? A review. Water Res..

[65] Zhou K., Zhang Y., Jia X. (2018). Co-cultivation of fungal-microalgal strains in biogas slurry and biogas purification under different initial CO2 concentrations. Sci. Rep..

[66] Kahn A., Oliveira P., Cuau M., Leão P.N. (2023). Incorporation, fate, and turnover of free fatty acids in cyanobacteria. FEMS Microbiol. Rev..

[67] Bhattacharya A., Mathur M., Kumar P., Prajapati S.K., Malik A. (2017). A rapid method for fungal assisted algal flocculation: critical parameters & mechanism insights. Algal Res..

[68] Spoof L., Jaakkola S., Važić T., Häggqvist K., Kirkkala T., Ventelä A.M., Kirkkala T., Svirčev Z., Meriluoto J. (2020). Elimination of cyanobacteria and microcystins in irrigation water—effects of hydrogen peroxide treatment. Environ. Sci. Pollut. Control Ser..

[69] Liu M., Shi X., Chen C., Yu L., Sun C. (2017). Responses of microcystis colonies of different sizes to hydrogen peroxide stress. Toxins.

[70] Saadi R., Saadi Z., Fazaeli R., Fard N.E. (2015). Monolayer and multilayer adsorption isotherm models for sorption from aqueous media. Kor. J. Chem. Eng..

[71] Ziane S., Bessaha F., Marouf-Khelifa K., Khelifa A. (2018). Single and binary adsorption of reactive black 5 and Congo red on modified dolomite: performance and mechanism. J. Mol. Liq..

[72] Robati D. (2013). Pseudo-second-orders kinetic equations for modeling adsorption systems for removal of lead ions using multi-walled carbon nanotube. J Nanostructure Chem.

