# Supplementary material for: Removal of cyanobacterial harmful algal blooms (HABs) from contaminated local park lake using Ganoderma lucidum mycelial pellets
Source: Heliyon. 2024 Dec 15;11(1):e41205. doi: 10.1016/j.heliyon.2024.e41205 (PMC11730225; doi:10.1016/j.heliyon.2024.e41205)
Supplement: Multimedia component 1 [file mmc1.docx]

Removal of Cyanobacterial Harmful Algal Blooms (HABs) from Contaminated Local Park Lake using *Ganoderma lucidum* Mycelial Pellets

Zarimah Mohd Hanafiah^1,2^, Anggita Rahmi Hafsari^1^, Malini Elango^1^, Zul Ilham^3^, Febri Doni^4^, Wan Hanna Melini Wan Mohtar^2*^, Yusufjon Gafforov^5^, Yong Jie Wong^6^, Khairul Nizam Abdul Maulud^2^, Nor Hidayah Ismail^7^, Mohd Yusmiaidil Putera Mohd Yusof^8*^, Wan Abd Al Qadr Imad Wan-Mohtar^1,8^*

^1^ Functional Omics and Bioprocess Development Laboratory, Institute of Biological Sciences, Faculty of Science, Universiti Malaya, Kuala Lumpur 50603, Malaysia; zarimahhanafiah@um.edu.my (Z.M.H); malinielango0205@gmail.com (M.E.); anggitarahmi@uinsgd.co.id (A.R.H); qadyr@um.edu.my (W.A.A.Q.I.W-M.)

^2^ Department of Civil Engineering, Faculty of Engineering and Build Environment, Universiti Kebangsaan Malaysia (UKM), Bangi 43600, Malaysia; hanna@ukm.edu.my (W.H.M.W.M); knam@ukm.edu.my (K.N.A.M)

^3^ Biomass Energy Laboratory, Faculty of Science, Institute of Biological Sciences, Universiti Malaya 50603 Kuala Lumpur, Malaysia; ilham@um.edu.my (Z.I)

^4^ Department of Biology, Faculty of Mathematics and Natural Sciences, Universitas Padjadjaran, Jatinangor, West Java 45363, Indonesia; febri@unpad.ac.id (F.D)

^5^ Central Asian Center for Development Studies, New Uzbekistan University, 100000, Tashkent, Uz-bekistan y.gafforov@newuu.uz (Y.G)

^6^ Department of Bioenvironmental Design, Faculty of Bioenvironmental Sciences, Kyoto University of Advanced Science, Kyoto 606-8501, Japan; wong.yongjie@kuas.ac.jp (Y.J.W)

^7^ Research Grant Management Division, Department of Research Management, Universiti Malaya, 50603 Kuala Lumpur, Malaysia nor_hidayah@um.edu.my (N.H)

^8^ Institute of Pathology, Laboratory and Forensic Medicine (I-PPerForM), Universiti Teknologi MARA Selangor, Sungai Buloh, Selangor, Malaysia, yusmiaidil@uitm.edu.my (M.Y.P.M.Y)

***** Correspondence: qadyr@um.edu.my (W.A.A.Q.I.W-M); hanna@ukm.edu.my (W.H.M.W.M); yusmiaidil@uitm.edu.my (M.Y.P.M.Y)

**FIGURE S1.** ANOVA posthoc (pH)


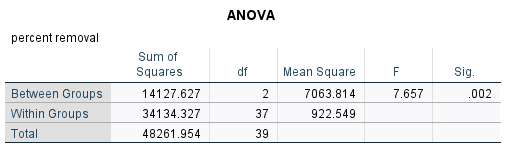


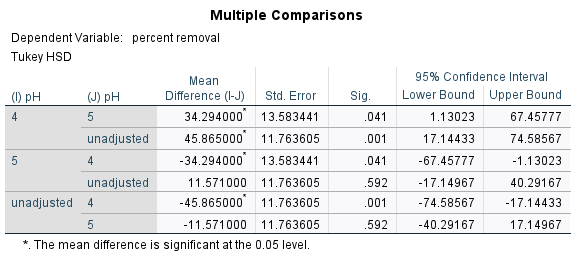


**FIGURE S2.** ANOVA posthoc (inoculum percentage)


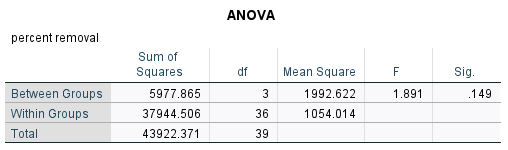


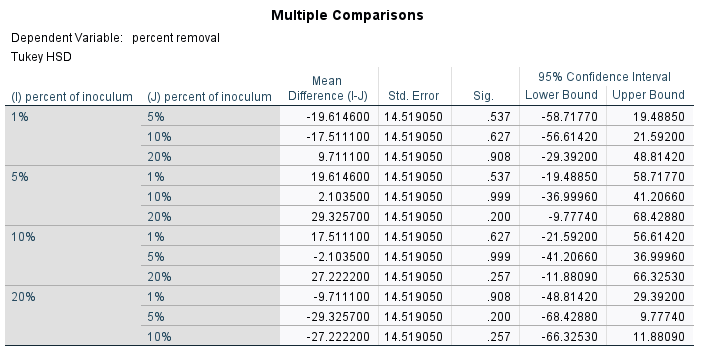


**TABLE S1.** Percentage removal of algal by *Ganoderma lucidum* at different pH

| Treatment  time (hour) | *G. lucidum*  (unadjusted pH) | | *G. lucidum*  pH4 | | *G. lucidum*  pH5 | | ctrl  (unadjusted pH) | | ctrl  pH4 | |
| --- | --- | --- | --- | --- | --- | --- | --- | --- | --- | --- |
| 0 | 0.00 | 0.00 | 0.00 | 0.00 | 0.00 | 0.00 | 0.00 | 0.00 | 0.00 | 0.00 |
| 6 | 0.00 | 4.35 | 2.56 | 12.50 | -20.00 | 4.35 | 25.00 | 16.00 | -10.71 | -15.79 |
| 24 | -5.00 | 13.04 | 30.77 | 42.50 | -5.00 | -8.70 | 21.43 | 24.00 | -39.29 | -57.89 |
| 30 | -40.00 | -34.78 | 71.79 | 60.00 | -10.00 | 4.35 | -7.14 | 0.00 | -82.14 | -121.05 |
| 48 | -125.00 | -69.57 | 56.41 | 62.50 | 5.00 | 26.09 | -53.57 | -8.00 | -57.14 | -84.21 |

**TABLE S2.** Percentage removal of algal by *Ganoderma lucidum* for 96 hours

| Treatment time (hour) | *G. lucidum* | | ctrl | |
| --- | --- | --- | --- | --- |
| 0 | 0.00 | 0.00 | 0.00 | 0.00 |
| 6 | 2.56 | 12.50 | -3.70 | 12.00 |
| 24 | 30.77 | 42.50 | 7.41 | 0.00 |
| 30 | 71.79 | 60.00 | 3.70 | -16.00 |
| 48 | 56.41 | 62.50 | 0.00 | -20.00 |
| 54 | 66.67 | 65.00 | 0.00 | -20.00 |
| 72 | 69.23 | 60.00 | 3.70 | -16.00 |
| 78 | 64.10 | 70.00 | -11.11 | -20.00 |
| 96 | 51.28 | 65.00 | -3.70 | -12.00 |

**TABLE S3.** Percentage removal of algal by *Ganoderma lucidum* at different inoculum volume (1%, 5%, 10% and 20%)

| Treatment time (hour) | 1% of *G. lucidum* | | 5% of *G. lucidum* | | 10% of *G. lucidum* | | 20% of *G. lucidum* | |
| --- | --- | --- | --- | --- | --- | --- | --- | --- |
| 0 | 0.00 | 0.00 | 0.00 | 0.00 | 0.00 | 0.00 | 0.00 | 0.00 |
| 6 | 2.56 | 3.87 | 67.35 | 65.37 | 56.1 | 55.36 | -57.5 | -16.87 |
| 24 | 30.77 | 38.78 | 75.51 | 70.70 | 60.98 | 62.17 | 50.00 | 49.56 |
| 30 | 71.80 | 76.86 | 59.18 | 63.14 | 70.73 | 73.15 | 70.00 | 66.46 |
| 48 | 56.41 | 55.90 | 65.31 | 66.52 | 68.29 | 65.25 | 30.00 | 48.18 |
